# Supplementary material for: MetaRibo-Seq measures translation in microbiomes
Source: Nat Commun. 2020 Jun 29;11:3268. doi: 10.1038/s41467-020-17081-z (PMC7324362; doi:10.1038/s41467-020-17081-z)
Supplement: Supplementary file 10 — Supplementary Data 7 [file 41467_2020_17081_MOESM10_ESM.zip › File2/Confidence_High_Taxonomy/88860_out.krona.html]

Javascript must be enabled to view this page.

members
magnitude
magnitudeUnassigned
count
unassigned
taxon
rank

88860\_out

3

superkingdom
3
2

1239
2
phylum

186801
2
class

186802
2
order

541000
family
1

946234
1
genus


SRS078665\_contig\_number\_contig-100\_76.76909
292800
1
species

family
1
31979

1
genus
1485

species
1
649724

SRS017433\_contig\_number\_9113

1
phylum
1117

order
1
1161

1
family
1162

genus
1
56106

species
1
142864

SRS075716\_contig\_number\_contig-100\_10062.10063
